# Use of Gene Transfer Vectors in Lymphedema Treatment: A Systematic Review

**DOI:** 10.7759/cureus.5887

**Published:** 2019-10-10

**Authors:** Antonio J Forte, Daniel Boczar, Maria T Huayllani, Sarah A McLaughlin, Sanjay Bagaria

**Affiliations:** 1 Plastic Surgery, Mayo Clinic Florida - Robert D. and Patricia E. Kern Center for the Science of Health Care Delivery, Jacksonville, USA; 2 Surgery, Mayo Clinic Florida - Robert D. and Patricia E. Kern Center for the Science of Health Care Delivery, Jacksonville, USA

**Keywords:** gene transfer vectors, gene expression, lymphedema, microsurgery, lymphovenous bypass, treatment

## Abstract

Different delivery mechanisms have been proposed in the literature for targeted therapies in the treatment of lymphedema. They vary from simple and direct injection to sophisticated induction of gene expression in a targeted tissue. We conducted a systematic review of publications assessing the use of viral vectors for gene transfer in lymphedema treatment. We hypothesized that viral vectors are an effective way to deliver targeted therapy in lymphedema treatment. We conducted a comprehensive systematic review of the published literature on targeted therapies associated with lymphedema surgery using the PubMed database. Eligibility criteria excluded papers that reported use of viral vectors for other medical conditions. Abstracts, presentations, reviews, meta-analyses, and non-English language articles were also excluded. From 21 potential articles found in the literature, fourteen fulfilled study eligibility criteria. Positive outcomes in terms of lymphangiogenesis were seen. The viral vectors used included adenovirus and recombinant adeno-associated virus. Most of the genes expressed were growth factors, but expression of dominant-negative transforming growth factor-β1 receptor-II or Prox1 was also proposed. Five studies targeted genetic expression on lymphedema tissue, five on transplanted lymph nodes, two on skeletal muscle, and one on adipose-derived stem cells. Publications assessing use of viral vectors for gene transfer in lymphedema treatment demonstrated that it is an effective mechanism of delivering targeted therapies. However, to date, all studies were experimental and further studies must be performed before translating these therapies into clinical practice.

## Introduction and background

Lymphedema is a chronic lymphatic condition that affects 140 to 200 million people in the world [1, 2]. It is estimated that one in every six patients undergoing surgical treatment for solid tumors will develop lymphedema. Affected patients can present with limb edema and dysfunction, pain, and skin ulceration. Interestingly, the majority of patients develop clinical manifestation only months after the lymphatic lesion, causing tissue fibrosis and demonstrating the relevance of further inflammatory steps [3-5].

Development of targeted therapies has attracted the interest of investigators around the world. Those therapies have been proposed in two main groups: targeted therapies modulating inflammation, such as those that control Th2-inflammatory responses [6-8], and targeted therapies inducing lymphangiogenesis, such as vascular endothelial growth factor (VEGF) C or adipose-derived stem cells (ADSCs) [9-11]. However, translation of some of these therapies into clinical practice raises concerns for increased risk of metastasis in oncologic patients [10].

Different delivery mechanisms for targeted therapies have been proposed in the literature. They vary from simple and direct injection to sophisticated induction of gene expression in a targeted tissue. The latter is possible through the use of viral vectors, such as adenovirus (short-term gene expression) and adeno-associated virus (long-term gene expression), encoding specific human genes [12]. We conducted a systematic review of publications assessing the use of viral vectors for gene transfer in lymphedema treatment. We hypothesize that viral vectors are an effective way to deliver targeted therapy in lymphedema treatment.

## Review

Materials and Methods

Search Strategy

Two reviewers (Daniel Boczar, Maria Huayllani) conducted independent searches using the PubMed database without timeframe limitations, initially through title and abstract screening, then by full-text review. Disagreements regarding article identification and final selection for inclusion of the literature were resolved by another reviewer (Antonio Forte). The search was conducted using the follow keywords: (((((((Adenovirus) OR Adenoviral) OR Adenovirally) OR Adenoviridae) OR Adenoviruses) OR Gene transfer vector)) AND ((Lymphedema) OR Breast Cancer Lymphedema). The bibliographies of those studies fulfilling eligibility criteria were also examined, looking for articles not present in our initial search. This study followed the guidelines outlined in the Preferred Reporting Items for Systematic reviews and Meta-Analyses (PRISMA).

Selection Criteria

Eligibility criteria included studies reporting data on the use of viral vectors for gene transfer in lymphedema treatment. Therefore, we excluded papers that reported use of viral vectors for other medical conditions. Abstracts, presentations, reviews, meta-analyses, and non-English language publications were also excluded.

Data Extraction and Processing

Extracted data included study year, country, study type, viral vector, targeted tissue, gene expressed, and key findings. Data extraction from articles, tables, and figures was performed by two reviewers (Daniel Boczar, Maria Huayllani), with the accuracy of data entry confirmed by an additional reviewer (Antonio Jorge Forte).

Results

Study Characteristics

**Figure 1:**
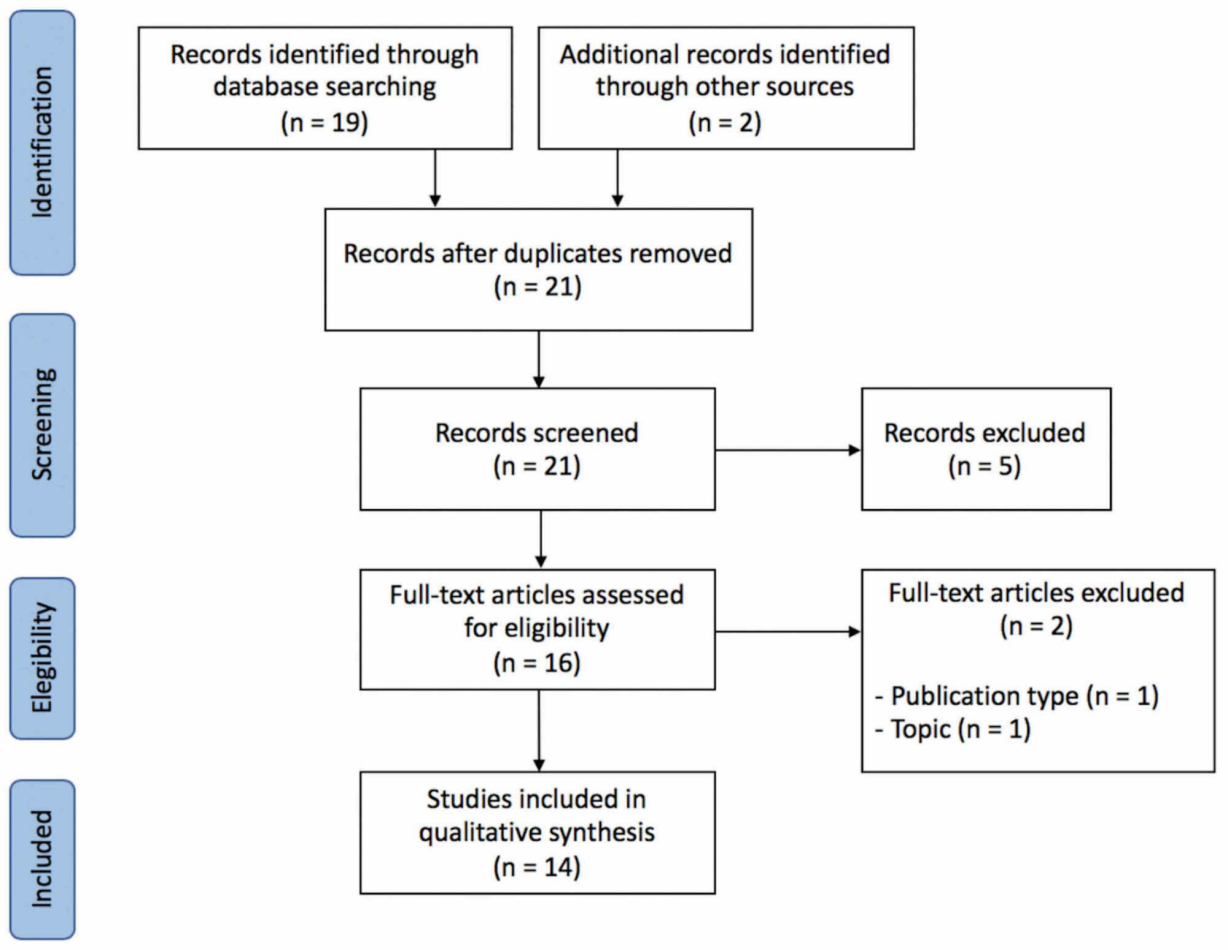
Preferred Reporting Items for Systematic Reviews and Meta-Analyses (PRISMA) diagram.

From 21 potential articles found in the literature, fourteen fulfilled study eligibility criteria (Figure 1, Table 1). Use of viral vectors for gene transfer in lymphedema treatment was described in experimental studies conducted in Finland (12/14), the United States (1/14), and China (1/14), with the first publication in 2001 [13]. Viral vectors used included adenovirus, recombinant adeno-associated virus, and lentivirus. Most experiments induced lymphangiogenesis through gene transfer of growth factors, but one used dominant-negative transforming growth factor (TGF)-β1 receptor-II and another used Prox1 (homeobox transcription factor for lymphatic endothelial cells differentiation). The most common transferred gene was VEGFC, but other genes were proposed, including VEGFD, VEGFC156S, and CCBE1. Five studies targeted genetic expression on lymphedema tissue, five on transplanted lymph nodes, two on skeletal muscle, and one on ADSCs.

 

**Table 1:** Summary of the Studies Abbreviations: AAV, Adeno-Associated Virus; ADSC, Adipose-Derived Stem Cells; LNT, Lymph Node Transfer.

Author	Year	Country	Study type	Model	Viral Vector	Target tissue	VEGF-C	VEGF-165	VEGF-189	VEGF-D	VEGF-A	CCBE1	Prox-1	TFG-Beta 1 receptor II
Karkkainen et al [13]	2001	Finland	Experimental	Mice	Adenovirus and AAV	Lymphedema	x							
SAARISTO et al [14]	2002	Finland	Experimental	Mice	Adenovirus and AAV	Lymphedema	x							
SAARISTO et al [15]	2002	Finland	Experimental	Mice	AAV	Lymphedema		x						
SAARISTO et al [16]	2004	Finland	Experimental	Mice	Adenovirus	Lymphedema	x	x						
Tammela et al [17]	2007	Finland	Experimental	Mice	AAV	Lymphedema	X	x	x					
Tammela et al [18]	2007	Finland	Experimental	Mice	Adenovirus	LNT	x			x				
Anisimov et al [12]	2009	Finland	Experimental	Mice	AAV	Skeletal muscle	x			x				
Lahteenvuo et al [19]	2011	Finland	Experimental	Pigs	Adenovirus	LNT	x			x				
Yan et al [20]	2011	USA	Experimental	Mice	Adenovirus	ADSCs								x
Honkonen et al [21]	2013	Finland	Experimental	Pigs	Adenovirus	LNT	x							
Jeltsch et al [22]	2014	Finland	Experimental	Mice	AAV	Skeletal muscle						x		
Tervala et al [23]	2015	Finland	Experimental	Mice	Adenovirus	LNT	x	x		x	x			
Visuri et al [24]	2015	Finland	Experimental	Pigs	Adenovirus	LNT	x	x						
Deng et al [25]	2017	China	Experimental	In vitro	Lentivirus	ADSCs							x	

Targeting Lymph Node Transplant

Studies have proposed viral vector encoding growth factors targeting lymph node transplant, including two studies comparing the therapeutic effects of VEGF-C and VEGF-D. Tammela et al. conducted a study on mice using adenovirus to express VEGF-C and VEGF-D in transplanted lymph nodes [18]. They noticed that both growth factors induced lymphangiogenesis compared to controls (lacZ β-galactosidase), in which transplanted lymph nodes regressed. Lymph nodes that had VEGF-C gene transfer joined with the pre-existing lymphatic network through afferent and efferent connections. Moreover, they were able to trap human lung carcinoma cells, subcutaneously injected during the experiment, in higher rates compared to controls [18]. Lahteenvuo et al. conducted an experiment on pigs, delivering adenovirus to encode VEGF-C and VEGF-D on transplanted lymph nodes [19]. Their results also demonstrated that VEGF-C has greater lymphangiogenic effects compared to controls. Moreover, they noticed that VEGF-D expression increased seroma, while VEGF-C expression did not [19]. 

Other studies assessed the therapeutic effect of other growth factors in lymphedema treatment. Tervala et al. compared the differences between therapeutic effects of growth factors on transplanted lymph nodes, using adenovirus to express genes of VEGF-C, VEGF-D, VEGF-C156S, and VEGF-A [23]. They noticed that VEGF-C provided the highest therapeutic results. Nonetheless, lymphangiogenesis was also greater for VEGF-D compared to controls, and better lymph node survival was seen for VEGF-D and VEGF-C156S [23]. Vissuri et al. conducted a study on pigs comparing the therapeutic effects of VEGF-C and VEGF-C156S delivered through adenovirus on transplanted lymph nodes [24]. Although both growth factors induced lymphangiogenesis, both lymphangiogenesis and lymph node preservation were greater with VEGF-C [24].

Differences in therapeutic effect between delivery to various locations in transplanted lymph nodes was also an object of interest. Honkonen et al. conducted a study on pigs to assess whether the location in which adenovirus encoding of VEGF-C gene was delivered in transplanted lymph nodes affected outcome [21]. Compared to control (saline), injection of adenovirus encoding VEGF-C at the intranodal and perinodal regions were able to induce lymphangiogenesis better and preserve the transplanted lymph node. However, they noticed that intranodal delivery presented the adverse effect of macrophage accumulation. Therefore, they postulated that perinodal delivery was preferable for future studies [21].

Targeting Skeletal Muscle

Some studies have proposed the use of gene transfer targeting skeletal muscle cells. Anisimov et al. assessed the therapeutic effects of long-term transgene expression of VEGF-C and VEGF-D in mice skeletal muscles, using adeno-associated virus [12]. They noticed that long-term expression of these two growth factors in skeletal muscles were able to generate new functional lymphatic and blood vessels, which could translate to better lymphatic drainage [12]. Similarly, Jeltsch et al. conducted a study on mice, delivering adeno-associated virus to encode CCBE1, which is part of the VEGF-C signaling pathway, and therefore, essential for lymphangiogenesis [22]. They noted that gene expression of CCBE1 in mice skeletal muscles increased lymphangiogenesis; therefore, postulating its utility in lymphedema treatment [22].

Targeting ADSCs

Two groups have conducted experiments in lymphedema treatment using viral vectors to induce gene expression in ADSCs. Yan et al. conducted a study on mice using adenovirus encoding a dominant-negative TGF-β1 receptor-II, blocking TGF-β1 anti-lymphangiogenic effects in ADSCs stimulated or not with VEGF-C [20]. They noticed that lymphangiogenic effect of VEGF-C was potentiated through genetic blockage of TGF-β1 [20]. Deng et al. conducted an in vitro study to assess the effects of lentiviral vectors encoding Prox1 (essential for lymphatic endothelial cells differentiation) in ADSCs to induce their differentiation into lymphatic endothelial cells [25]. They noticed that ADSCs overexpressing Prox1 started to present specific lymphatic endothelial cell markers, including podoplanin and VEGF receptor 3. Moreover, those cells formed tube-like structures which resembled lymphatic vessels [25].

Targeting Lymphedema Tissue

Several studies have proposed use of gene transfer targeting lymphedema tissue. Karkkainen et al. conducted a study on mice, intradermally injecting adenovirus and recombinant adenovirus into the right ear to encode VEGF-C [13]. They noted that virus-mediated VEGF-C therapy generated functional lymphatic vessels [13]. Saaristo et al. conducted three studies on mice, injecting viral vectors to encode growth factors. In one experiment, they overexpressed VEGF-C through adenovirus and adeno-associated virus in the skin and respiratory tract of athymic nude mice, demonstrating that VEGF-C induced dose dependent changes in blood vessels, such as vascular leak [14]. In another study using viral vectors to encode VEGF-C156S (VEGFR-3-specific mutant form of VEGF-C), they demonstrated that long-term expression of VEGF-C156S promoted functional cutaneous lymphatic vessels with normal morphology [15]. In their most recent study, Saaristo et al. [16] compared the therapeutic effect of virally expressed VEGF-C and VEGF-C156S, demonstrating that despite the transient expression of the adenoviral encoding gene, mice treated with VEGF-C demonstrated a stable lymphatic vessel function within a two-month follow-up. Tammela et al. conducted a study on mice, comparing the therapeutic effects of VEGF-C and VEGF-heparin-binding domain chimeras (ie, VEGF-165 and VEGF189) encoded by adenovirus or adeno-associated virus [17]. They noticed that VEGF-heparin-binding domain chimeras activated VEGF-C receptors and stimulated lymphangiogenesis. Interestingly, compared to VEGF-C, different patterns of lymphangiogenesis were noticed. The chimeras promoted lymphangiogenesis along tissue borders, supporting lymphatic vessels with a larger lumen compared to VEGF-C [17].

Discussion

Research on targeted therapies in lymphedema treatment has been increasing considerably over the years. Delivery of growth factors in lymphedema treatment can be exogenous (recombinant human growth factors) or endogenous, through gene expression induced by viral gene transfer. In this systematic literature review, we have shown that the use of viral vectors for gene transfer in lymphedema treatment was effective in several different experimental studies. Most studies had the rationale to induce lymphangiogenesis through gene transfer of growth factors, and VEGF-C was the most common gene transferred in the experiments. Moreover, different types of tissues and cells were targeted in the experiments, including lymphedema tissue, transplanted lymph nodes, skeletal muscles, and ADSCs. None of the studies described unintended consequences of injecting viral vectors, but experiments had a limited follow-up period. To our knowledge, this study is the first systematic literature review assessing the use of viral vectors for gene transfer in lymphedema treatment.

We recognize several limitations to our study typical to systematic reviews, including the potential for bias in interpreting the data reported in each study. Moreover, we excluded non-English language publications, such as a study by Lu et al. from China, who conducted an experiment in which adenoviral VEGF-C was delivered to a transferred lymph node [26]. Nonetheless, this systematic review reports a valuable summary of the scientific evidence regarding the use of viral vectors for gene transfer in lymphedema treatment, which can guide future studies to advance the field. Further studies on large animals and phase 1 clinical trials are necessary to ensure the safety of gene transfer viral vectors in lymphedema treatment.

## Conclusions

The publications assessing the use of viral vectors for gene transfer in lymphedema treatment demonstrated that it is an effective mechanism of delivering targeted therapies. To date, all experiments pursued gene transfer, and most used growth factors to induce lymphangiogenesis. Although encouraging outcomes were seen, all studies to date have been experimental, so further studies are necessary to translate those therapies into clinical practice.
